# Liposarcoma of the Spermatic Cord: A Case Report

**DOI:** 10.1155/2011/197584

**Published:** 2011-09-25

**Authors:** M. S. Papageorgiou, G. Dadakas, K. Donev

**Affiliations:** ^1^Department of Surgery, Larnaca General Hospital, Larnaca, Cyprus; ^2^Department of Urology, Larnaca General Hospital, Larnaca, Cyprus

## Abstract

Liposarcomas are malignant tumors derived embryologically from mesodermal tissues. An unusual site of presentation is the spermatic cord, presenting as an inguinal or scrotal mass. We report a rare case of a liposarcoma of the spermatic cord, mimicking a testicular tumor. The patient was operated, and an orchidectomy, including the tumor, was performed. To our knowledge, there are about 185 similar cases reported in the literature.

## 1. Introduction

Liposarcomas are malignant tumors derived embryologically from mesodermal tissues. They represent the most common soft-tissue sarcomas, and they can occur in any part of the body that contains fatty tissue. Although lipomas of the inguinal canal, derived from the spermatic cord, are a usual finding in many operations on the inguinal canal or the scrotum, when they are excluded from diagnosis in cases of spermatic cord masses, the possibility of malignancy is as high as 56% [1]. Liposarcomas of the spermatic cord usually begin to grow directly below the external inguinal ring, so when the tumors reach a large size, they present as scrotal rather than inguinal mass. We report a rare case of a liposarcoma of the spermatic cord, mimicking a testicular tumor, which was treated surgically. To our knowledge, there are about 185 similar cases reported in the literature, and we report our case as well, since it was a rare finding during an everyday routine operation, a case that can happen to every specialist at anytime.

## 2. Case Presentation

A 73-year-old Caucasian male presented with a slow-growing left scrotum mass in the Outpatient Department of the Urology Department of Larnaca General Hospital. He had a personal history of hypertension and hypercholesterolaemia treated with medication. The mass was first noticed by the patient 18 months ago, and it was continuously growing ever since, without the patient seeking medical advice before. In physical examination, the mass was large, hard and was interpreted as a testicular tumor. Ultrasonography indicated a left testicular tumor, and further investigation was advised. The patient was admitted, and a surgical intervention for biopsy and possible orchidectomy was decided. In surgery, the mass was found to be not of testicular origin, but it was growing from the spermatic cord, descending into the scrotum, around the testicle. The mass was measuring about 10 cm, it had a yellowish lipoma-like texture, but in the center of the mass, a hard round tumor was palpated. A left orchidectomy was decided and performed, including the mass, which was sent for pathology. The patient's postoperative course was uncomplicated, and he was discharged on the 2nd postoperative day. Pathology report revealed a liposarcoma measuring 10 × 8 × 7 cm, with evidence of myxoid degeneration, with mitotic activity (0–3 mitoses/10 HPF). Immunohistochemical analysis was negative to HMB45, Melan A, CD34, CD31, HHF35, Desmin, SMA, and ceratines AE1/AE3, but was positive to S100.

## 3. Discussion

Liposarcomas of the spermatic cord are extremely rare malignant tumors, representing 3%–7% of all paratesticular sarcomas [2, 3]. They are classified in 4 histology subtypes (well differentiated, myxoid, pleomorphic, and dedifferentiated) [3]. These tumors occur more frequently in adults rather than children [4], and although cases aging 16–90 years old are reported, the mean age at presentation is 56 years [5]. Liposarcomas usually present as slow-growing masses of the inguinal canal or the scrotum, mimicking testicular or epididymal tumors or inguinal hernias, and they are often diagnosed either intra- or postoperatively. Ultrasonography is often nonspecific for this type of lesions, and the most accurate preoperative examinations in order to place a diagnosis are CT or MRI [2, 4]. In suspicious cases, in which preoperative imaging techniques are not adequate for the diagnosis, preoperative biopsy may be needed, in order to have a definite diagnosis [2]. The treatment of choice for liposarcomas of the spermatic cord is radical orchidectomy with a high ligation of the spermatic cord [6], with an excellent prognosis, but these tumors seem to have a tendency towards local recurrence (*≈*25%) [7]. In the literature, several cases of various histologic subtypes are reported, including myxoid degeneration, sclerosing or inflammatory types, pleomorphic, and even cases with cartilaginous metaplasia [8–13]. Well-differentiated tumors usually have no metastatic potential, although the rate of metastases is high in undifferentiated tumors, usually through hematological route to lungs and bones [2, 8]. Chemotherapy and/or radiotherapy are controversial and are only limited in cases of metastatic tumors or in cases following incomplete resection.

In conclusion, liposarcomas of the spermatic cord represent a rare type of tumors, which are often misdiagnosed preoperatively. To our knowledge, there are about 185 cases reported in the literature, and because most of the cases are in the form of case reports or cases series, there is not a clear view regarding the physical course and the proper treatment and prognosis of the disease.
